# Synthesis of Silver Nanostructures by Multistep Methods

**DOI:** 10.3390/s140405860

**Published:** 2014-03-25

**Authors:** Tong Zhang, Yuan-Jun Song, Xiao-Yang Zhang, Jing-Yuan Wu

**Affiliations:** 1 School of Electronic Science and Engineering, Southeast University, and Key Laboratory of Micro-Inertial Instrument and Advanced Navigation Technology, Ministry of Education, Nanjing 210096, China; E-Mails: songyuanjunwf@163.com (Y.-J.S.); zxycom@163.com (X.-Y.Z.); wwjjyy92@163.com (J.-Y.W.); 2 Suzhou Key Laboratory of Metal Nano-Optoelectronic Technology, Suzhou Research Institute of Southeast University, Suzhou 215123, China; 3 School of Chemistry and Chemical Engineering, Southeast University, Nanjing 211189, China

**Keywords:** reductant, nanoparticle etching, composited nanostructures, shape control, plasmonic sensors

## Abstract

The shape of plasmonic nanostructures such as silver and gold is vital to their physical and chemical properties and potential applications. Recently, preparation of complex nanostructures with rich function by chemical multistep methods is the hotspot of research. In this review we introduce three typical multistep methods to prepare silver nanostructures with well-controlled shapes, including the double reductant method, etching technique and construction of core-shell nanostructures. The growth mechanism of double the reductant method is that different favorable facets of silver nanocrystals are produced in different reductants, which can be used to prepare complex nanostructures such as nanoflags with ultranarrow resonant band bandwidth or some silver nanostructures which are difficult to prepare using other methods. The etching technique can selectively remove nanoparticles to achieve the aim of shape control and is widely used for the synthesis of nanoflowers and hollow nanostructures. Construction of core-shell nanostructures is another tool to control shape and size. The three methods can not only prepare various silver nanostructures with well-controlled shapes, which exhibit unique optical properties, such as strong surface-enhanced Raman scattering (SERS) signal and localized surface plasmon resonance (LSPR) effect, but also have potential application in many areas.

## 1. Introduction

Recently, control over silver nanoparticle morphologies has received considerable attention due to their potential applications in catalysis [1,2], biological and chemical sensors [3–8] and surface-enhanced Raman spectroscopy [9–11]. Actually, for over a thousand years ago, people have used silver as an antibacterial and disinfectant as recorded in a book on Chinese herbal medicine called *Compendium of Materia Medica*. In decades past, synthesis of silver nanostructures has been an active research area because of their excellent optical properties such as surface-enhanced Raman scattering (SERS) [12] and plasmonic resonance, which strongly depend on size, shape and composition [13–15]. Particularly, the shape control is vital to improve the optical properties of the resulting nanostructures. Therefore, many groups have devoted their efforts to exploring ways to prepare well-defined silver nanostructures in high yields. Among the numerous methods, the chemical method is thought to be the most popular. Xia's group [16–20] successfully synthesized various well shape-controlled silver nanostructures by the polyol process in ethylene glycol (EG) through varying the precursor concentration, molar ratio of the stabilizer and silver ions, reaction temperture and addition of helper agents. However, during these processes, reaction conditions are harsh, and are complex or difficult to control. For example, the reactant injection rate is critical for the shape of the final products, which makes the procedure difficult to operate. In addition, the reaction atmosphere is very important for the synthesis of the desired silver nanostructures [21], because in the presence of oxygen, twinned particles may be etched preferentially because of higher reactivity. Conversely, without oxygen, there was no oxidation etching to dissolve twinned particles leading to the formation of silver nanowires. Helper agents such as stabilizers and some ionic species also play a great role in the shape and size control of silver nanostructures. Polyvinyl pyrrolidone (PVP) is one of common stabilizers used in the synthesis of silver nanostructures. Sun's group [22–24] produced uniform silver nanowires by taking advantage of the selectively adsorption on the (100) facets of PVP. If PVP is absent or added in low amounts, the main products are mainly nanospheres. Meanwhile, they found that silver nanoparticles with irregular morphology were formed in the presence of lower molecular weight PVP. In our previous work [25], we also demonstrated the shape of silver nanostructures can be controlled by varying the molecular weight. Wiley *et al.* [26] explored the role of different ions in the shape-controlled synthesis process. Results indicated that the addition of Cl^−^, Br^−^ and Fe^3+^ promote the formation of silver nanocubes, right bipyramids and nanowires, respectively. Without fine control of reactant conditions and growth process, the obtained silver nanostructures are always obtained in low yield accompanied by large amounts of by-products. In these cases, the post processing, such as low rotation-rate centrifugation or special separation technique to purify products, is usually indispensable. Therefore, it is highly desirable to develop a reliable and facile method for the synthesis of silver nanostructures in high yield with well shape and size control.

Besides the chemical reduction method, photochemical synthesis is also applied in the preparation of silver nanostructures. Compared with chemical reduction methods, the size distribution of the nanoparticles obtained is uniform. Stabilized intermediates can be obtained, which is difficult to carry out by other methods. Monodisperse silver decahedrons with finely tuned sizes were produced by Pietrobon and Kitaev [15] using a novel photochemical technique. Moreover, the high symmetry and uniform size distribution causes narrow plasmon peaks with a tuned range and significant enhancement of Raman signature. Machulek Junior *et al.* [27] prepared silver nanoprisms in the presence of PVP via extended irradiation of nanospheres solution with visible light. Jin *et al.* [28] synthesized silver nanoprisms through plasmon excitation and explored the growth process in detail by analyzing TEM photographs. Although the size of nanoprisms was controlled by adjusting light wavelength, no size enlargement occurred with addition of precursors which is definitely different from thermal methods.

Although great achievements have been made in the preparation of silver nanostructures during the past decades, some issues still cannot be solved using conventional methods by simply changing the precursor concentrations or reaction temperature and time, such as tuning the size of nanoparticles over a wide range. Yang *et al.* [29] reviewed two kind of unconventional methods including lithography and template-based methods to fabricate metallic nanostructures with large area. Different nanostructures with uniform shapes and sizes and different composition can be produced flexibly by using templates such as ion-track etched polymer membranes or nanoporous anodized alumina membranes. Although post-processing is relative complex, which may affect the purity of products, these unconventional methods are surely efficient and powerful. Therefore, in the review we will introduce some other unconventional multistep methods which can synthesize shape-controlled and novel silver nanostructures including the double reductants method, etching technique and construction of core-shell nanostructures. Jones *et al.* [30] covered a number of templates for the preparation of plasmonic nanostructures including solution-phase templates, porous templates and surface mask templates. They have mentioned part of the etching technique and core-shell nanostructures in their review. However, we reviewed these unconventional methods from three perspectives following different rules. The double reductant method is based on different favorable facets of silver nanocrystals produced in different reductants. The etching technique involves the use of an etchant to selectively remove nanoparticles so that nanostructures can be obtained with shape control. The mechanism of construction of core-shell nanostructures is epitaxial growth from core seeds. The optical properties of these nanostructures can be finely tuned corresponding to the shape and size control leading to wide range of potential applications.

## 2. Double Reductant Method

It is known that different reductants can offer different reducibility, which plays an important role in shape control of nanostructures. Moreover, favorable facets of nanocrystals are determined by the reductants used. Some reductants prefer to promote growth of (100) facets, while others prefer to (111) or (110) facets. Therefore, complex nanostructures or nanostructures which are not easy to be prepared using one-step methods can be obtained by choosing different reductants in each step leading to desired nanostructures.

### 2.1. N,N-dimethylformamide (DMF) and EG

**Figure 1.**
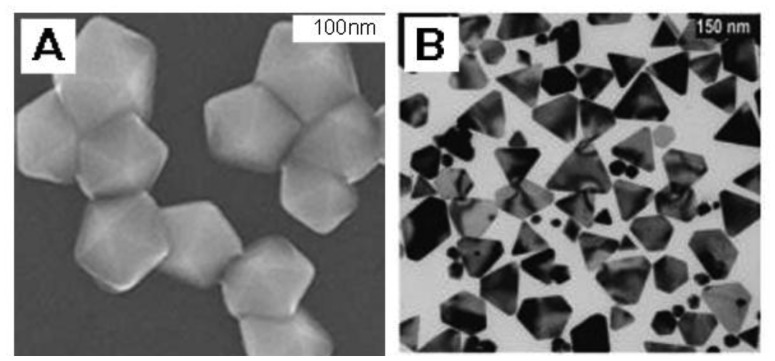
SEM and TEM images of silver nanoparticles with different morphologies, prepared by reduction of Ag^+^ ions by DMF, in the presence of PVP: (**A**) decahedrons (reprinted with permission from [37]. Copyright (2006), Elsevier); (**B**) prisms (reprinted with permission from [35]. Copyright (2009) WILEY-VCH Verlag GmbH & Co. KGaA, Weinheim, Germany).

**Figure 2.**
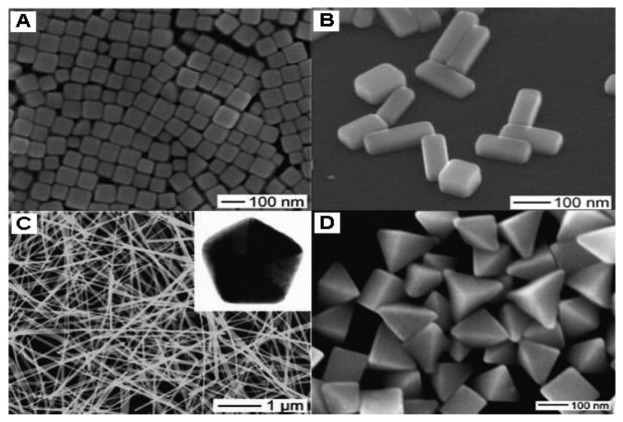
Electron microscopy images of single-crystal Ag nanocrystals: (**A**) nanocubes prepared in ethylene glycol with PVP as a capping agent; (**B**) nanobars prepared in ethylene glycol in the presence of PVP and Br^−^; (**C**) nanowires prepared in ethylene glycol in the presence of PVP; (**D**) bipyramids prepared in ethylene glycol in the presence of PVP. (reprinted with permission from [41]. Copyright (2007) American Chemical Society).

DMF is a well-known organic solvent as well as an active reductant under suitable condition which has been demonstrated [31]. Liz-Marzán's group first employed DMF to reduce AgNO_3_ for the preparation of silver nanostructures which paved a new way for shape control [32]. In their later works, they successfully synthesized nanospheres [33], nanoprisms [34,35] and nanowires [36] via reduction of AgNO_3_ by DMF in the presence of PVP. In addition, Gao *et al.* [37] prepared silver decahedrons in high yield with PVP as stabilizer in DMF. Tsuji *et al.* [38] provided new information on the growth of decahedrons and icosahedrons in DMF through a stepwise route. Lu *et al.* [39] realized the finely tuned size of nanoplates from 20 to 50 nm by varying the molar ratio of PVP/DMF. In DMF the main products are triangular and hexagonal plates, decahedra and icosahedra having (111) facets, as shown in Figure 1, while in EG the typical products are cubes, right-triangular, bipyramids and pentagonal rods and wires [9–11,14–16,20,40,41] having (100) facets, as shown in Figure 2.

**Figure 3.**
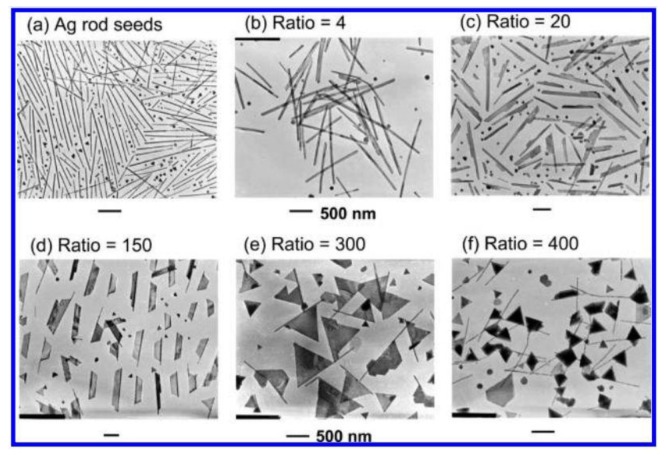
TEM images of Ag rod seeds and flag types of Ag nanostructures prepared in DMF at various [AgNO_3_]_2_/[AgNO_3_]_1_ ratios (reprinted with permission from [43]. Copyright (2010) American Chemical Society).

**Figure 4.**
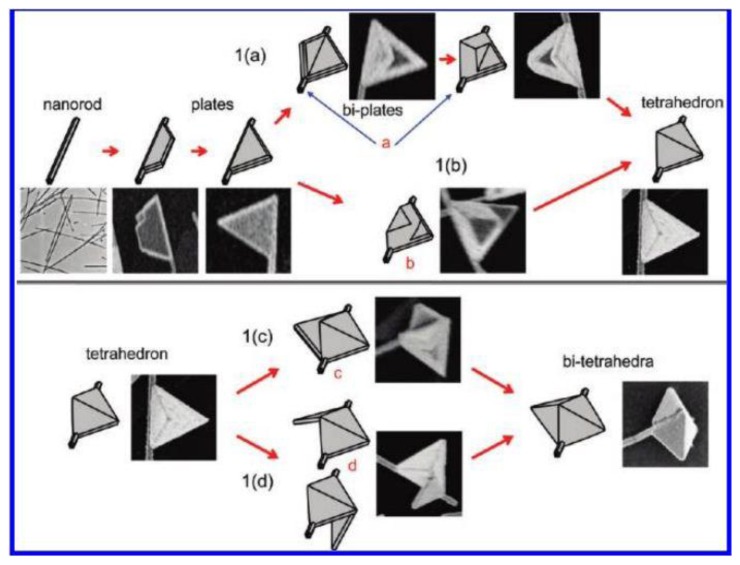
Growth mechanisms of silver nanoflags from silver nanorods in DMF (reprinted with permission from [43]. Copyright (2010) American Chemical Society).

Based on this, Tsuji *et al.* [42] opened a new way to synthesize octahedral and triangular having (111) facets which were hard to obtain via previous methods. The production process was to prepare cubic and right bipyramids having (100) facets by reducing AgNO_3_ in EG first, then the shape of these nanoparticles were transformed into octahedral and triangular shapes in DMF. The shape evolution from cubic and right bipyramidal to octahedral and triangular is assigned to the different facets of Ag nanocrystals in EG and DMF. Sequently, they fabricated Ag nanoflags using a similar two-step method by preparing Ag nanorods as seeds in EG and growing nanoflags in DMF [43]. The following [AgNO_3_]_1_ and [AgNO_3_]_2_ stand for the concentration of AgNO_3_ in the first and second step respectively. In the first step silver nanorod as shown Figure 3a was prepared by reduction of AgNO_3_ in EG in the presence of PVP, and then was dispersed in DMF as a seed solution. In the second step, seed solution was added into a DMF solution containing AgNO_3_ and PVP. With the increase of the ratio of [AgNO_3_]_2_/[AgNO_3_]_1_, different structures were observed (Figure 3b–f). They explained the growth mechanism of silver nanoflags from nanorods in detail as shown in Figure 4. In the second step, a trapezoid plate formed from the side surface of silver nanorods, and then grew into a triangular twin plate. Next the triangular flags transformed into tetrahedral flags via two routes. As route 1a in Figure 4 shows, another triangular flag formed in another side of the silver nanorod, and then grew to the other one until the two triangular flags connected together leading to tetrahedral flags. The other route (route 1b in Figure 4) is that two triangular pyramidal structures grew from the former triangular flags and finally evolved into tetrahedral flags. If [AgNO_3_]_2_ is too high, the tetrahedral flags can continue to grow into twin tetrahedral flags which can be seen from route 1c in Figure 4. Therefore, the double reductant method gives a new way to manufacture octahedral, triangular and novel silver nanostructures in high yield and well-controlled in size and shape which are difficult to obtain via one-step methods.

### 2.2. Sodium Borohydride(NaBH_4_) and Ascorbic Acid (AsA)

Aside from EG and DMF, NaBH_4_ is also frequently used as a reductant to synthesize nanosized Ag colloids. Many groups have studied the reduction of Ag^+^ ion in NaBH_4_ which is usually used with some stabilizer, such as citrate and PVP. Zhang *et al.* [44] explored a new way to control the size and shape of Ag nanospheres by changing the rate of nucleation and growth in the reduction of Ag^+^ ion by NaBH_4_ together with trisodium citrate (Na_3_CA) and PVP. In the absence of Na_3_CA, nanospheres cannot be prepared because Ag nucleuses are immediately protected by PVP as soon as they form leading to the anisotropic growth of Ag nanoparticles due to selective adsorption of PVP on the (100) facets. When PVP was absent, the products were also nanospheres with larger size. The results indicate that the role of Na_3_CA is to promote the reduction of Ag^+^ ion into nanospheres. On the other hand, PVP can promote nucleation and prevent the aggregation of nanoparticles. In our previous work [45], we prepared branched silver nanowires and nanomeshworks by using both NaBH_4_ and Na_3_CA in the presence of PVP. Silver seeds were prepared firstly, and then exposed under light leading to aggregation and were thus welded together, so the final morphology was decided by the concentration of PVP. Wojtysiak [46] also studied the important effect of citrate on the reduction of Ag^+^ ions by NaBH_4_. Stable Ag nanoclusters only appeared when citrate was added, otherwise reduction of Ag^+^ ion by NaBH_4_ under the conditions with citrate led to a precipitate at the bottom of solution. Citrate is a stabilizer as well as a reductant. In this regard, Yi *et al.* [47] used NaBH_4_ and Na_3_CA as a double reductant system to prepare Ag nanoplates via a multistage procedure. In the first stage, Ag nanoplates were prepared as seeds with NaBH_4_ and Na_3_CA. Then these as-prepared seeds evolved into larger nanoplates with tunable sizes of 40 nm to 260 nm by adding different volumes of Na_3_CA solution. Each addition of Na_3_CA solution and seed solution was named one stage, the size of the nanoplate increased without change of shape, and the SPR peak shifted red. To get triangular nanoplates with satisfactory size distribution and yield, Yang *et al.* [48] applied a novel double reductant method which consists of three steps. In the first step, silver nanospheres were prepared through reduction of AgNO_3_ by NaBH_4_ in the presence of Na_3_CA. In the second step, sodium dodecyl sulfate (SDS) was added as stabilizer into the as-prepared silver nanoparticle colloid and then citrate-stabilized silver nanospheres were converted into SDS-stabilized silver nanospheres. In the last step, silver nanoplates were formed under the reduction of SDS-stabilized silver nanospheres by citrate and aged in NaCl solution. When SDS-stabilized silver nanospheres aged in NaCl solution for one week, they began to transformed into nanoplates. Three weeks later, high-yield silver nanoplates with larger size were formed. In their further experiments, if only NaBH_4_ was used as reductant in the presence of SDS, no silver nanoplates appeared, which indicated citrate is essential to reduce SDS-stabilized silver nanospheres in the third step.

**Figure 5.**
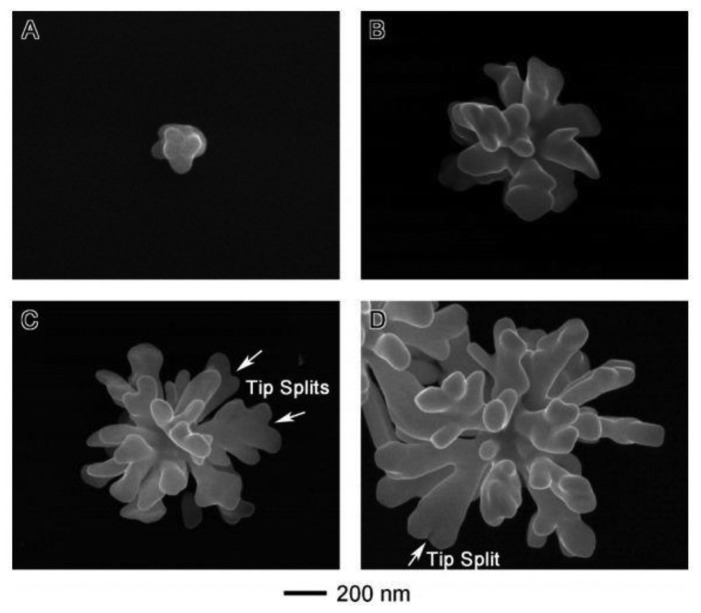
SEM images of the silver particles sampled at different reaction times (**A**) 3–8 s; (**B**) 20–30 s; (**C**) 1 min; and (**D**) 5 min. Arrows indicate examples of tip splits (reprinted with permission from [52]. Copyright (2008) American Chemical Society).

**Figure 6.**
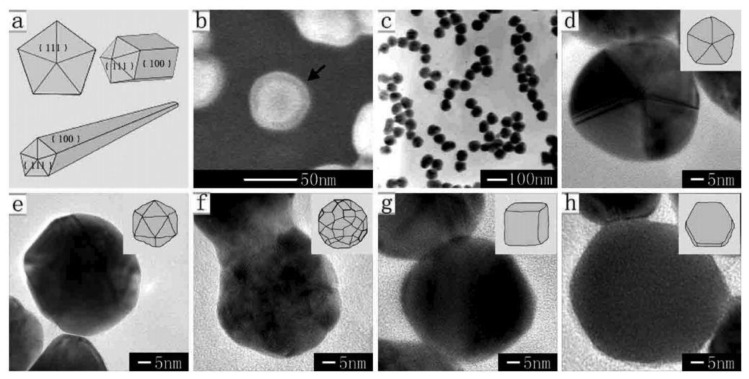
Inhibition of the anisotropic growth of Ag crystals: (**a**) sketch of the twinned decahedron, rod- and wire-shaped crystals; (**b**) FE-SEM image, indicating the adsorption of PVP on the surface of a Ag particle; (**c**) TEM image of as-prepared 50 nm Ag particles; (**d**–**h**) HR-TEM images of as-prepared 50 nm Ag particles for the particles with the shape of decahedron, icosahedron, quasi-sphere, cube, and hexagon, respectively; insets are sketches of the corresponding structures (reprinted with permission from [56]. Copyright (2010) American Chemical Society).

More recently, a method of using AsA to prepare unique silver nanostructures such as flower-like and string forms has been developed [49]. Zheng *et al.* [50] got silver dendrites when they reduced AgNO_3_ by AsA in the presence of cetyltrimethylammonium bromide (CTAB) and SDS. Lou *et al.* [51] also found that hyperbranched silver nanostructures can be obtained by reduction of AgNO_3_ by AsA with Na_3_CA. Based on these researches of hyperbranched silver nanostructures mentioned above, Wang *et al.* [52] explored the growth mechanism of hyperbranched silver nanostructures in AsA. In their opinion, as Figure 5a shows, multifaceted particles with bulbous tips were formed from the reduction of AgNO_3_ by AsA in the initial stage of reaction, which are easy to aggregate in AsA [53]. After 20–30 s, silver atoms were deposited on the bulbous ends of the seeds resulting in the growth of the branched particles (Figure 5b. Figure 5c,d illustrate that with longer reaction time, the size continues to increase and secondary branches began to grow from the first branches that originate from the seeds. Moreover, tips can be observed at the ends of branches. In addition to AsA which acts as a key factor in the formation of branched structures, stabilizers such as starch [54] and PEO-PPO-PEO tri-block copolymer [55] also play an important role in controlling the morphology of silver nanostructures. Besides, a method of using AsA to prepare a series of silver nanostructures by reducing AgCl in aqueous solution has been developed by Chen *et al.* [56]. Their key idea for growing different morphologies silver nanoparticles is to adjust amount of sodium hydroxide (NaOH). Because the reduction power of AsA is related to pH which can be varied by adding NaOH, silver nanoparticles seeds of different sizes were prepared firstly in the presence of AsA and NaOH, and then AsA/AgCl/PVP/NaOH solution system was added to control the shape. Taking 50 nm silver seeds as an example, Figure 6 shows FE-SEM images of silver nanostructures obtained from as-prepared seeds in AsA with different dosages of NaOH. Furthermore, they follow the formula (1) to predict the size of the product:

(1) $$\text{D}=\text{d}\times \sqrt[{3}]{\frac{\text{N}+\text{n}}{\text{n}}}$$

**Figure 7.**
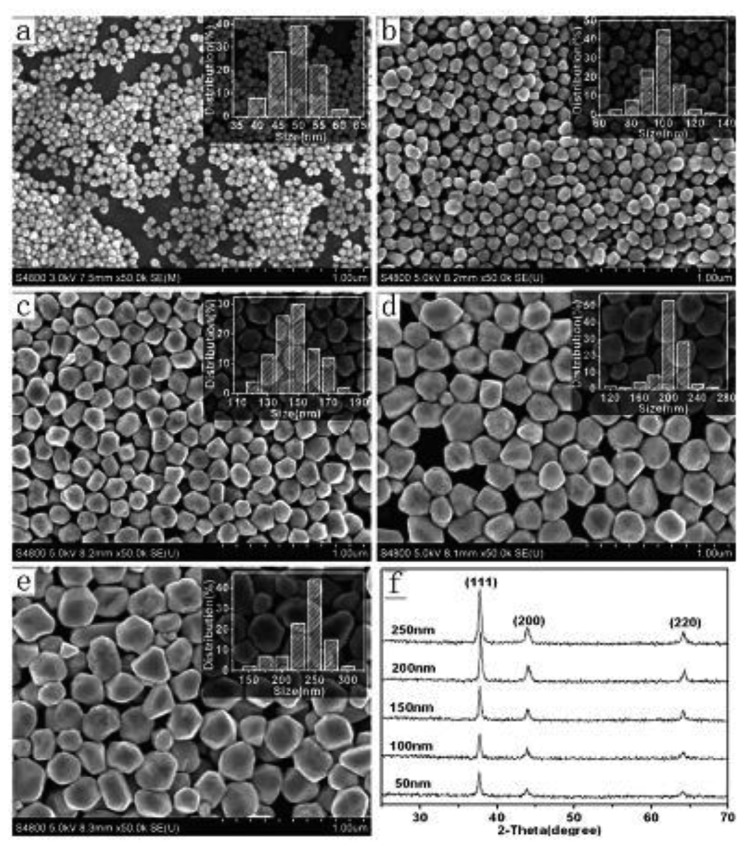
FE-SEM images of 50 nm Ag seeds (**a**) and as-prepared products of quantitative size-controlled and size-designed synthesis with sizes of (**b**) 100 nm; (**c**) 150 nm; (**d**) 200 nm; and (**e**) 250 nm. Reaction times are 16 h, insets are the corresponding size-distribution histograms; (**f**) shows the XRD patterns corresponding to (**a**–**e**) (reprinted with permission from [56]. Copyright (2010) American Chemical Society).

In the formula, D is the average size of product, d is the average size of seeds, and N and n are the molar amount of AgCl and seeds respectively. They fixed the amount of AgCl, and used 50 nm silver nanoparticles as seeds, so in theory if they want obtain the products with 100, 150, 200, 250 nm, the (N+n)/n should be 8, 27, 64, and 125 nm respectively. As shown in Figure 7, the experiment results are consistent with the prediction.

Above all, we can get a conclusion which is that the products tend to be nanospheres of small sizes in NaBH_4_ which are usually used as seeds for further growth of silver nanocrystals, while silver nanoparticles are easy to aggregate together in AsA. Samanta [57] presented a two-step method to produce silver nanodiscs and triangular nanoplates with both NaBH_4_ and AsA as double reductants. They prepared Ag nanoparticles in NaBH_4_ in the first step. When these nanoparticles used as seeds with different amounts were added into AgNO_3_ solution in AsA, silver nanodiscs and triangular nanoplates formed, respectively. A high yield of triangular nanoplates can be obtained in lower amount of seed solution which provides a new path to synthesize triangular nanoplates.

## 3. Etching Technique

Recently, shape and size control as well as novel nanostructures which can increase SERS enhancement have attracted more attention. The optical properties of nanostructures can be tuned by varying their sizes, therefore it is vital to provide a way to control the size. In addition, these nanostructures with sharp horns such as nanoflowers or nanostars can focus the electromagnetic field on their tips leading to significant SERS effect. The etchant technique can not only control shapes and sizes of nanostructures, but also creates novel nanostructures with hot spots or hollow nanostructures.

### 3.1. Hydrogen Peroxide (H_2_O_2_)

Early in the 1950s, H_2_O_2_ is used to etch pits in metals, which plays an important role of dislocations in determining or affecting the mechanical properties of crystalline materials [58]. The standard potential in the H_2_O_2_—water couple is dependent on the pH value of the solution. In acidic solutions:

(2) $$\begin{array}{cc} H_{2}O_{2}+2H^{+}+2{\text{e}}^{-}\to 2H_{2}O & E^{0}=1.763V \end{array}$$

In alkaline solutions:

(3) $$\begin{array}{cc} H_{2}O_{2}+2{\text{e}}^{-}\to 2OH^{-} & E^{0}=0.867V \end{array}$$

Because the potentials are higher than that of *Ag*^+^/*Ag* (*E*^0^=0.7996*V*) [59], H_2_O_2_ can be used as an effective etchant to dissolve metallic silver. Very recently, etching techniques are used for controlling the shapes of Ag nanoparticles because some nanostructures are not easily prepared in high yield or mono-dispersed sizes.

**Figure 8.**
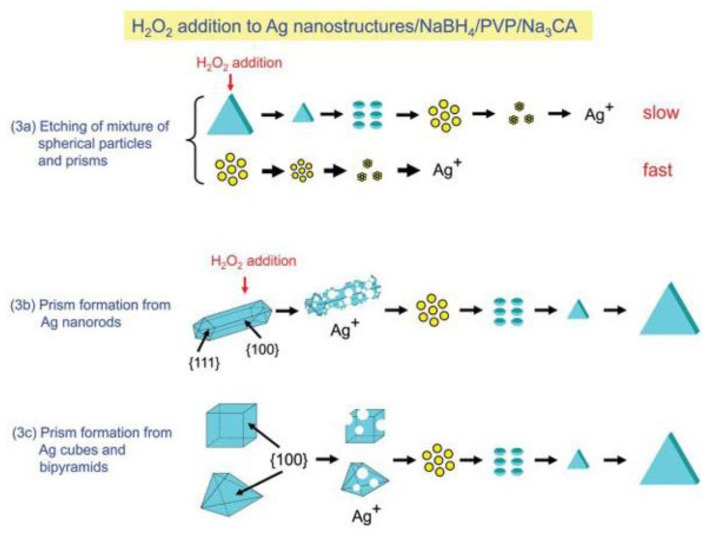
Growth mechanisms of ag nanostructures prepared from mixtures of spherical nanoparticles and prisms and nanorods, cubes, and bipyramids (reprinted with permission from [66]. Copyright (2012) American Chemical Society).

Among these various shapes, Ag nanoprisms and triangulars have attracted intense interest due to their unique optical properties and related applications. Generally, two main methods have been developed to prepare Ag nanoprisms, including the photochemical method and chemical reduction method. Compared with the photochemical method [60–63], the chemical reduction method is considered a popular and a simple route, however, this method cannot provide relative uniform size distributions. M'etraux and Mirkin [64] reported a new chemical reduction route to prepare Ag nanoprisms which is a great breakthrough in the size control of silver nanostructures. In the process, in the absence of H_2_O_2_, the products were nanospheres, which indicated that H_2_O_2_ plays a critical role of Ag nanoprism formation. Sequentially, Zhang *et al.* [65] studied in detail the role of H_2_O_2_ in the same reaction system. They proposed that H_2_O_2_ can remove the relatively unstable nanoparticles in the nucleation stage and promote the formation of anisotropic structures, finally all metallic silver particles can be directly transformed into silver nanoplates regardless of size and shape, but if the concentration of H_2_O_2_ was too high, the obtained nanoprisms also would be etched and disappear. To further study the mechanism, Tsuji *et al.* [66] examined the relative etch rate of prism to sphere by monitoring the time-dependent SPR band of the mixture of spheres and prisms. Similarly, they used a flag type of Ag nanostructure which contained triangular plates and a pentagonal rod to examine the relative etch rate of prism to rod. The results were: *ν*_prism_ < *ν_sphere_* and *ν*_prism_ < *ν_rod_*. If either H_2_O_2_ or Na_3_CA is absent, the transformation from spheres to prisms will not happen and Na_3_CA must be added before H_2_O_2_. The major difference between the method and Zhang's is that the etching of metallic Ag nanostructures and reduction of Ag^+^ ion occur at the same time in the latter reaction process, but the former argued that the first step is complete dissolution of Ag nanowire to Ag+ ion and then reduction of Ag^+^ ion to Ag^0^. They also successfully prepared Ag nanoprisms from nanocubes and nanobipyramids. This essay made a great contribution to preparing high-yield Ag nanoprisms from different metallic Ag nanostructures. Figure 8 presents the growth mechanisms of silver nanoprisms from the mixture of spherical nanoparticles and prisms and nanorods, cubes, and bipyramids.

Recently, metallic hollow nanostructures such as cages [67–70] and frames [71,72] which have bigger surface area have aroused intense interest because they can broadly tune localized surface plasmon resonance (LSPR) and exhibit excellent optical, electronic and catalytic properties. Zhang *et al.* [73] explored a new method based on H_2_O_2_ etching to prepare Au nanoboxes. Because the standard reduction potential of AuCl_4_^−^/Au is higher than that of Ag^+^/Ag, Au can be replaced by Ag following Equation (4):

(4) $$3Ag(s)+AuCl_{4}^{-}(l)=Au(s)+3AgCl(s)+Cl^{-}(l)$$

**Figure 9.**
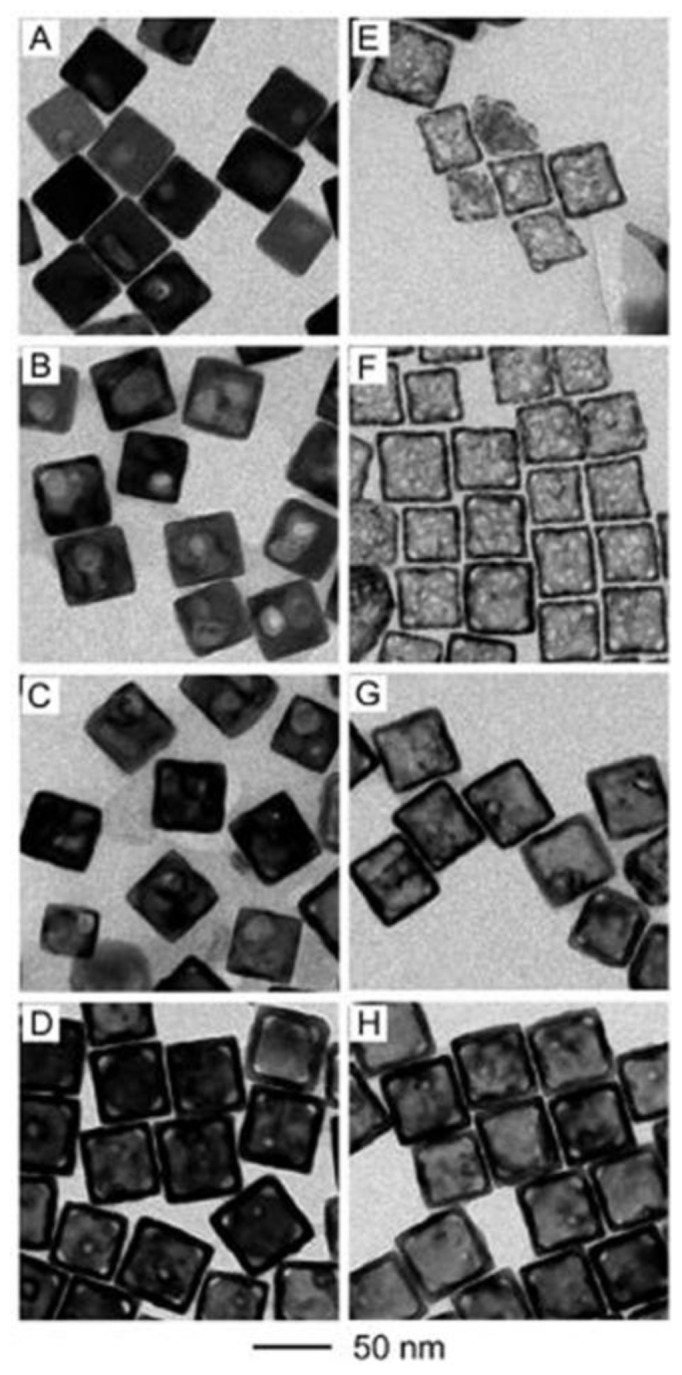
TEM images of Au -Ag nanoboxes before and after etching with an excess amount of H_2_O_2_: (**A**–**D**) the Au-Ag nanoboxes with their LSPR peaks being tuned to 460, 520, 675, and 745 nm, respectively, prior to the addition of H_2_O_2_; and (**E**–**H**) the corresponding Au-based nanocages after the addition of excess H_2_O_2_ (reprinted with permission from [73]. Copyright (2010) American Chemical Society).

Based on the chemical equation, silver nanoboxes can be transformed into gold nanoboxes. The synthesis procedure is to form Au-Ag alloy nanoboxes by titrating Ag nanocubes with aqueous HAuCl_4_. By controlling the amount of HAuCl_4_ aqueous, they can precisely control the thickness of nanoboxes via titrating HAuCl_4_ aqueous solution as presented in Figure 9a–d, causing the redshift of LSPR peaks. Then Ag atoms are removed from the nanoboxes and alloy walls via H_2_O_2_ etching. Figure 9e–h show the corresponding Au-based nanocages after the addition of excess H_2_O_2_. McEachran *et al.* [74] proposed a similar pathway to synthesize well-defined gold nanoframes from decahedral silver nanoparticals, but unlike Xia *et al.* they controlled the thickness of frames by adjusting the amount of deposited gold. Furthermore, they attempted to make similar nanoframes using other silver nanostructures. Results indicated that only nanostructures with (111) facets such as nanorods and icosahedra can be used for the formation of nanoframes. They argued that gold prefers to deposit on the (111) facets, whereas (100) facets are more reactive to cause nanocages or nanoshells. We believe that the etching technique is a facile method to prepare hollow nanostructures with well-controlled shapes and sizes.

**Figure 10.**
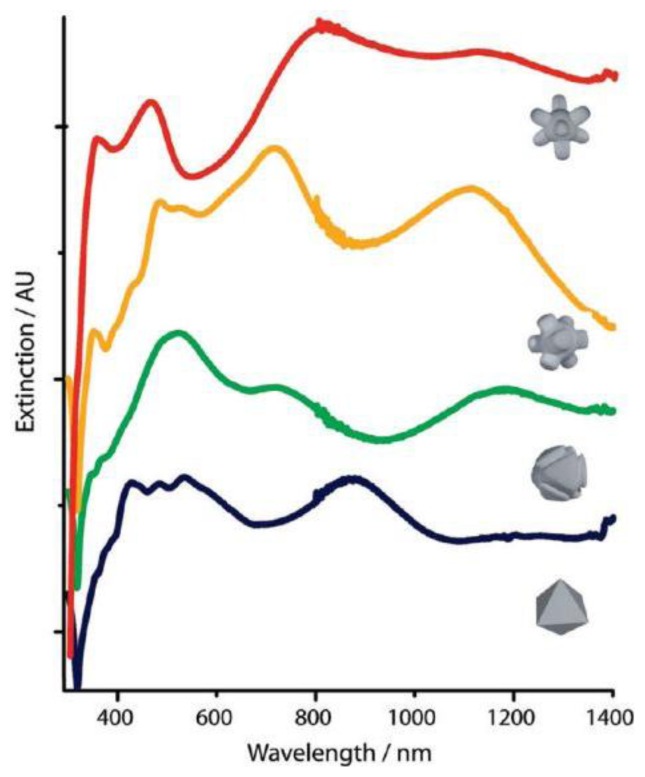
UV-vis-NIR spectra of different stages of the etching progress when starting from octahedral nanoparticles. Interestingly, as the etching progresses the octopod structures have LSPs with greater intensity in the red and near-infrared regions (reprinted with permission from [79]. Copyright (2010) American Chemical Society).

It is well known that nanostructures with sharp edges or gaps can enhance SERS intensity [75–78], therefore the etching technique can also be applied to prepare flower-like silver nanostructures or make the surface of nanocrystals rough. For example, Yang *et al.* [79] etched silver nanooctahedra by H_2_O_2_ and NH_4_OH mixtures. From Figure 10 we can see that the intensity of extinction significantly increases along with the etching progress and the octopod structures have LSPR with greater intensity in the near-infrared regions (red line in Figure 10). They presented a novel etching technique for synthesis of complex nanostructures which have broad range of resonance band and strong enhancement of SERS signals. Another method increasing the intensity of SERS is to fabricate nanostructures with hot spots. The definition of hot spots is the junctions or gaps between two or more closely spaced nanoparticles in which enormous electromagnetic enhancements are generated in contrast to individual particles [80]. The formation of hot spots has attracted much attention, including the theoretical aspects of electromagnetic enhancement factors in SERS [81] and experimental fabrication of nanostructures with hot spots [82–84]. In theory, Ag nanowire is an ideal nanostructure with large surface to be a SERS substrate, however, the smooth surface limits the ability to form localized surface plasmons. To solve the problem, Goh *et al.* [85] increased the number of SERS hot spots on the surface of silver nanowires by etching in H_2_O_2_ and NH_4_OH mixture resulting in a 10^4^-fold enhancement of SERS compared with that obtained by pre-etching. Some ways have been reported to attach nanoparticles onto nanowires [86–89], however, the enhancement of SERS is strongly related to the interaction between nanowires and nanoparticles rather than localized surface plasmons. It is asserted that the etching technique can be widely used for the fabrication of SERS substrates with potential applications in many areas.

### 3.2. Other Etchants

There are many other etchants applied to modify the morphology of silver nanoparticles, such as NH_4_OH or Fe(NO_3_)_3_. Sometimes NH_4_OH is used with H_2_O_2_, which has been discussed in Section 3.1., therefore we will not reintroduce it here. Lu *et al.* [90] found that NH_4_OH or Fe(NO_3_)_3_ play an important role in the formation of Au nanoboxes. The process consists two steps as follows: (1) preparation of Au/Ag alloy nanoboxes through depositing Au on the surface of Ag nanocubes; (2) removal of Ag from Au/Ag alloy nanoboxes via etching by NH_4_OH or Fe(NO_3_)_3_. After the formation of the Au nanobox, sequent addition of NH_4_OH or Fe(NO_3_)_3_ causes the transformation into nanocages and nanoframes. Another advantage of this method is that NH_4_OH or Fe(NO_3_)_3_ can selectively remove Ag from the walls of Au/Ag alloy nanoboxes leading to good control in the thickness of nanocages which is difficult to achieve by other methods.

## 4. Construction of Core-Shell Nanostructures

Although Ag nanostructures have received considerable attention because of their excellent optical properties and wide applications in many areas, people still attempt to develop novel nanostructures for higher requirements. The optical properties of nanostructures can also be tailored by controlling their elemental composition, as well as the internal and surface structures. Most recently, special attention has been paid to core-shell nanostructures because they provide a new system with tunable optical properties. In this case, core-shell nanostructures can be achieved through various methods which have been developed, such as chemical reduction method, nanosphere lithography (NSL) [91,92], photochemical method [93–96].

### 4.1. Au@Ag Core-Shell Nanostructures

Au@Ag core-shell nanostructures exhibit finely tuned LSPR and SERS properties based on the shape and size, and have potential applications in sensors [91,97–99] and catalysis [100], thus many attempts have been made to prepare these nanostructures.

The shape and size of the Au core is important for the evolution of Ag on Au nanoparticles. Many groups select Au nanorods as cores to construct the Au@Ag core-shell nanostructures to which excellent optical properties are ascribed. Liu *et al.* [101] described two different routes to coat the Au nanorods with silver for the first time. One way is to reduce AgNO_3_ by AsA on the surface of Au nanorods with sodium citrate used as stabilizer, but the repeatability of results is not good and it is difficult to take TEM pictures due to the high concentration of sodium citrate. In this regard, they chose PVP as stabilizer instead of sodium citrate. However, they found that Ag^+^ ions cannot be reduced in low pH solution, because the redox potential of AsA varied with pH until it was sufficient to overcome that of Ag. Therefore, when NaOH was added or AsA was replaced by citric acid which is a weak acid, Ag shells formed. The results indicated that with the increased amount of AgNO_3_ added, the thickness of the Ag shell increased and the blue-shift of the longitudinal plasmon mode of the nanorods was enhanced. Later on, Seo *et al.* [102] demonstrated that Ag nanorods can be grown directly from Au decahedrons and nanorods. Benefiting from the success of Seo in forming Au@Ag core-shell nanorods from Au decahedron seeds, we can get a deeper understanding of the growth mechanism of nanorods. In the initial stage, Ag^+^ ions were reduced and deposited on the decahedral seeds. Then, Ag nanocrystals grew along the longitudinal direction with slight changes along the lateral direction assigned to the selective adsorption of PVP on the (100) facets. The results correspond to the growth mechanism of Ag nanorods proposed by Xia *et al.* However, in the research of Xia *et al.* [103], the morphology of Ag nanoparticles were octahedrons grown from Au nanorods. They explained that Au@Ag core-shell nanorods were transition modes, when more Ag was deposited, (100) and (110) facets began to disappear and the nanorods finally evolved into octahedrons. In addition, they can easily tune the size of Ag octahedrons by using Au nanorods with different aspect ratios or controlling the amount of AgNO_3_ added to the reaction system. In the case of other Au nanorods as seeds, Sanchez-Iglesias *et al.* [104] also produced Ag octahedron shelsl from Au nanorods in a different way. Compared with the work of Xia *et al.* [103], they controlled the thickness of Ag shells from thin to thick not only by using different aspect ratios of Au nanorods, but also using methoxypoly(ethylene glycol)-thiol (mPEG-SH) as stabilizer which can bind at the Au nanorod tips to block the growth of Ag along the longitudinal direction leading to the formation of Ag octahedrons. Moreover, they demonstrated that the optical properties of these core-shell constructs can be finely tuned with the change of thickness of Ag shell and the Au@Ag core-shell octahedrons with thin shells showing optical properties similar to those of pure Ag octahedrons.

**Figure 11.**
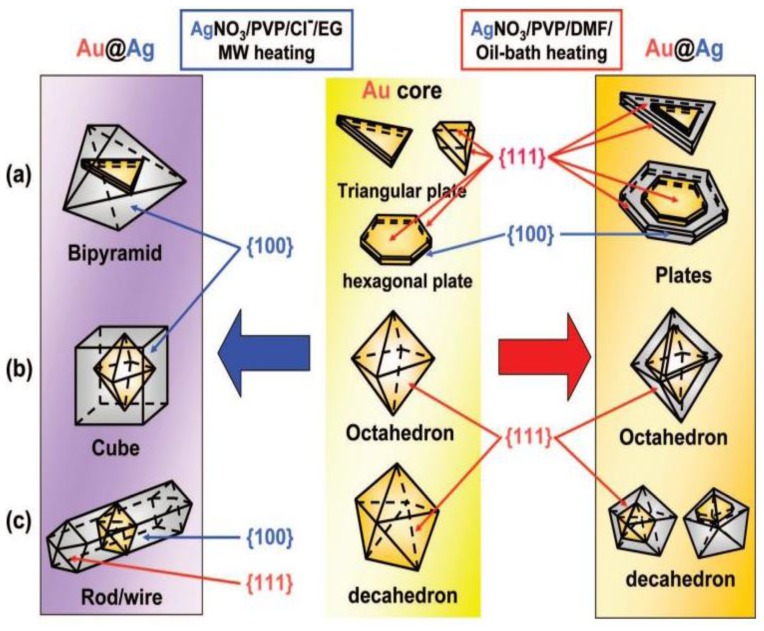
Schematic crystal structures of the Au cores and Au@Ag nanocrystals prepared in DMF by an oil-bath heating (right side) and in EG by a MW heating (left side) (reprinted with permission from [94]. Copyright (2008) American Chemical Society).

As with the above-mentioned Ag nanoprisms and triangular synthesized by H_2_O_2_ etching, additional seed-mediated growth method are often used to construct Au@Ag core-shell nanoprisms and triangulars. Xue *et al.* [96] reported a photochemical method to form Au@Ag core-shell nanoprisms. In their experiments, the role of Au seed was as a photocatalyst leading to the growth of Ag triangular shells which slightly depended on the shape of the Au cores. However, the morphology of Ag triangles can be easily tuned by changing the excitation wavelength and size of the Au core. In 2009, their group produced Au@Ag core-shell triangular bifrustums via chemical reduction route by AsA [100]. Compared with the photocatalyst they used, the sizes of Ag triangles were bigger and the triangular bifrustum became thicker with the increase of the addition amount of AgNO_3_, significantly affecting the SPR spectrum. Recently, Tsuji's group developed a combination of microwave and polyol reduction route to prepare Au@Ag core-shell triangles [94] as Figure 11 shown. In the first step, Au nanocrystals seeds were obtained by reducing HAuCl_4_ in EG in the presence of PVP under microwave heating conditions. In the second step, as-prepared Au seeds were added into a DMF solution of AgNO_3_ to form Ag shells in an oil bath. The method can prepare not only triangular Au@Ag core-shells, but also nanoplates, octahedrons and decahedrons depending on the shapes of the Au seeds. In addition, these Ag shell shapes are significantly different from those prepared in EG by microwave heating from the same Au seeds. The reason is the favorable facets of Ag nanostructures produced in EG and DMF are different, which has been discussed before. Using the microwave-polyol method, their group also prepared Au@Ag core-shell icosahedrons [105]. In 2012, they explored the growth mechanism of Au@Ag core-shell octahedra and decahedra using a similar method [106]. They argued that nanocrystal growth process mainly depends on the way of heating. Uniform Au@Ag core-shell octahedra and decahedra can be obtained under oil bath heating, while under fast microwave heating the Ag shell is not uniform. Above all, the microwave-polyol method provides a new and facile path for synthesize various Au@Ag core-shell nanostructures.

In addition to these Au@Ag core-shell nanostructures above, Ag nanocubes also receive much attention. Fan *et al.* [107] synthesized Au@Ag core-shell nanocubes by using Au octahedra as seeds in high yield for the first time. Later on, Ma *et al.* [108] described a simple method for preparing Au@Ag core-shell nanocubes from Au nanopheres. In this case, the edge lengths of the Ag nanobox can be finely tuned from 13 to 50 nm by controlling the amount of AgNO_3_ or Au seeds which were close to the calculated results, and the thickness of Ag shell can be controlled precisely from 1.2 to 20 nm because the seeds are isotropic and have uniform size. Moreover, they found that the effect of CTAC as capping agent on the formation of Au@Ag core-shell nanocubes was better than that of CATB.

### 4.2. Other Composited Nanostructures

According to Table 1, we can get that it is difficult to construct noble metal@Ag core-shell nanostructures except gold by epitaxial growth because these nanostructures must accord with three criteria listed as follows [107]: (1) the atomic radius of shell should be smaller than that of core which is easier to grow epitaxially; (2) the shell should have smaller bond dissociation in order to ensure stable growth; (3) the electronegativity of the shell should be smaller than that of core, or displacement reactions will occur. Therefore, a few core-shell nanostructures are prepared by the epitaxial growth method. Tsuji *et al.* [109] synthesized Ag@Cu alloy by reducing the mixture of AgNO_3_ and Cu(OAc)_2_·2H_2_O in EG, then they used Ag@Cu alloy to produce new Ag@Cu alloy Cu shells, which was consistent with the rules. Hu *et al.* [110] proposed thatb ZnO@Ag nanoflowers can be prepared by depositing zinc power on silver layers using a vapor transportation method under an oxygen atmosphere. Moreover, Ag@Pt bimetallic nanoparticles were prepared by Karthikeyan *et al.* [111] via a chemical reduction method.

## 5. Application

In the past decades, silver nanoparticles have been applied to many areas because of their excellent optical properties. The three methods introduced in this essay can not only control the shapes and sizes of silver nanostructures well, but also their LSPR properties, leading to more potential applications.

**Figure 12.**
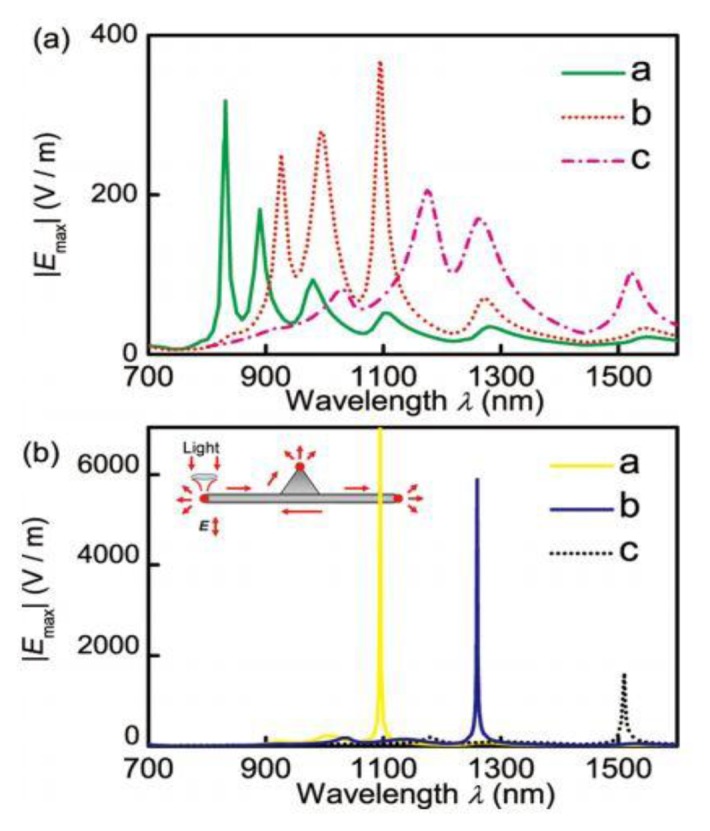
(**a**) Curves a–c plot the relationship between |Emax| and the wave-length for long nano-flags (L = 2 um) with W = 150, 200, and 275 nm, respectively; (**b**) Curves a–c show the relationship between |Emax| and the wavelength for long nano-flags (L = 2 um) with W = 210, 250, and 310 nm, respectively. The inset in (**b**) illustrates the position of the focused incident light spot in the simulation model for the excitation of the long nano-flags (reprinted with permission from [112]. Copyright (2012) AIP Publishing LLC).

**Figure 13.**
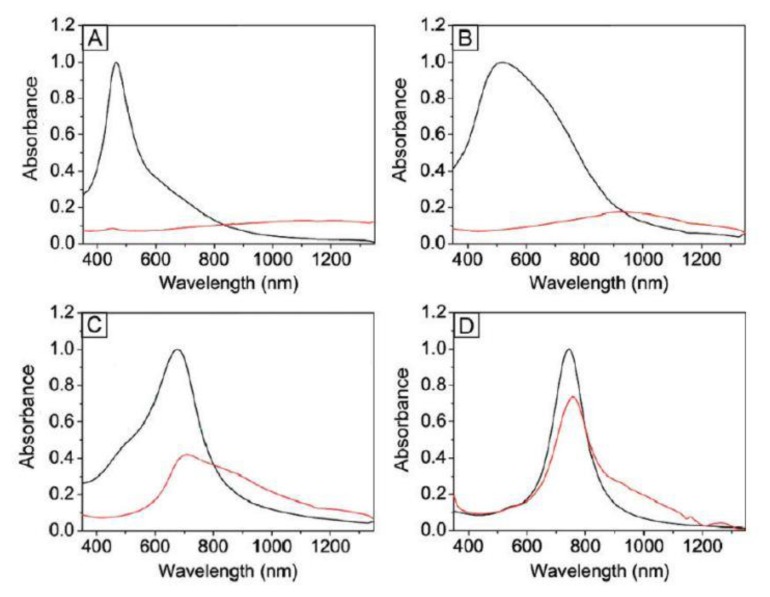
UV-vis-NIR spectra of the samples that correspond to the Au-Ag alloy nanoboxes and nanocages shown in Figure 5. The spectra in black are for the starting Au-Ag nanoboxes and the spectra in red are for the resulting Au-based nanocages after a reaction with an excess amount of H_2_O_2_. For the nanoboxes, the LSPR peaks were at (**A**) 460; (**B**) 520; (**C**) 675; and (**D**) 745 nm. The spectra were normalized to unity. For the nanocages, the LSPR peak was red-shifted to the near-infrared region for the first two samples of nanocages due to their extremely thin walls. The spectra were normalized by dividing the same factor to the corresponding spectra of the nanobox (reprinted with permission from [73]. Copyright (2010) American Chemical Society).

As mentioned in Section 1.1, Tsuji *et al.* have synthesized silver nanoflags which combine a nanowires and a triangular nanoplate by a double reductant method as shown in Figure 4. Zhang *et al.* [112] studied the plasmonic properties of silver nanoflags by theoretical simulation. In their simulations they investigate the spectral properties of silver nanoflags of different widths. It can be seen from Figure 12a that resonance spectra of silver nanoflags induced by SPP mode depends on the width of the nanoplate. When the width is tuned some special values as shown in Figure 12b, the spectrum has a single resonance band with an ultra-narrow bandwidth and significant electric-field enhancement. Therefore such a nanoflag can be used in spacer-based nanolasers. The plasmonic properties of porous of Au-Ag alloy nanocages were studied by the finite element method [113]. The results show that the plasmon mode can be tunable by changing the surface porosity. When a Au nanobox transforms into a nanocage, the plasmon mode shifts red, which means that larger surface porosity causes more redshift. The simulation result is demonstrated by Zhang *et al.* [73]. They prepared Au-Ag alloy nanoboxes by the H_2_O_2_ etching technique. In Figure 13 we see the absorption spectra of Au-Ag alloy nanoboxes (black line) and nanocages (red line). It can be seen that LSPR peaks of Au-Ag nanoboxes are red-shifted from 460, 520, 675 and 745 nm to 1210, 925, 790 and 758 nm, respectively, after the treatment with excess H_2_O_2_. Because the scattering and absorption of Au nanocages can be tailored by controlling the size and porosity, Chen *et al.* [114] functionalized Au nanocages with an antibody to cancer cells. In addition, they found Au nanocages have a photothermal effect when selectively attached to cancer cells, killing them by inducing localized heating. The remarkable LSPR properties of Au nanocages make them promising in the biomedical area.

**Figure 14.**
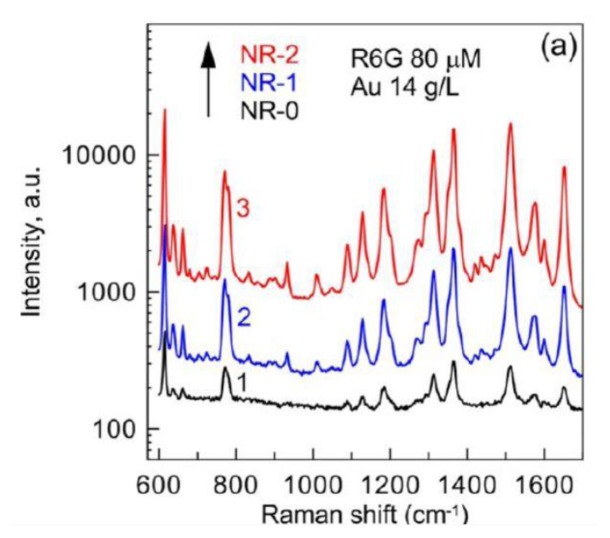
SERS spectra of R6G (1 μL, 80 μ M) deposited with NR-0 (1), NR-1 (2), and NR-3 (3) rods at a Au concentration of 14 g/L (reprinted with permission from [115]. Copyright (2013) American Chemical Society).

Au@Ag core-shell nanostructures have attracted considerable attention because of their improved properties compared with silver nanostructures or gold nanostructures, especially optical properties, which allows them to be used as SERS substrates. Generally, SERS substrates should be sensitive, stable and rapid. Khlebtsov *et al.* [115] investigated the SERS spectra of rhodamine 6G (R6G) by using Au@Ag core-shell nanorods as substrate. NR-0, NR-1 and NR-2 represent gold nanorods, Au@Ag core-shell nanorods with 1.05 nm and 2 nm silver shells, respectively. It can be seen from Figure 14 that the Raman signal of R6G with NR-1 was much higher than NR-0, and with the increase of thickness of silver shells, the intensity enhanced. They assumed that the SERS enhancement of Au@Ag core-shell nanorods was related to the electromagnetic field distribution. To confirm their assumption, they resorted to three-dimensional finite-difference time-domain (FDTD) simulations, which indicated that the hotspots near the end-to-end and end-to-side nanoparticle contacts caused the SERS enhancement of Au@Ag core-shell nanorods. We previously reported that gold nanoparticle thin films can be used as sensitive SERS substrates, because hot spots formed in the narrow gaps regions between neighboring nanoparticles can enhance the Roman signal [11]. This opinion was also proved by Costa *et al.* [116]. They prepared Au@Ag core-shell nanotubes with hotspots in the surface, which exhibited more excellent SERS properties than silver nanowires. It is well known that silver nanoparticles are easy to oxidize, so another challenge is how to keep the SERS substrate stable. Gute *et al.* [117] found that when silver dendrite was coated by gold and was used as SERS substrate, the stability was improved with a slight decrease of intensity of the SERS enhancement. Therefore, Au@Ag core-shell nanostructures can serve as ideal SERS substrates for chemical and biological detection processes.

**Figure 15.**
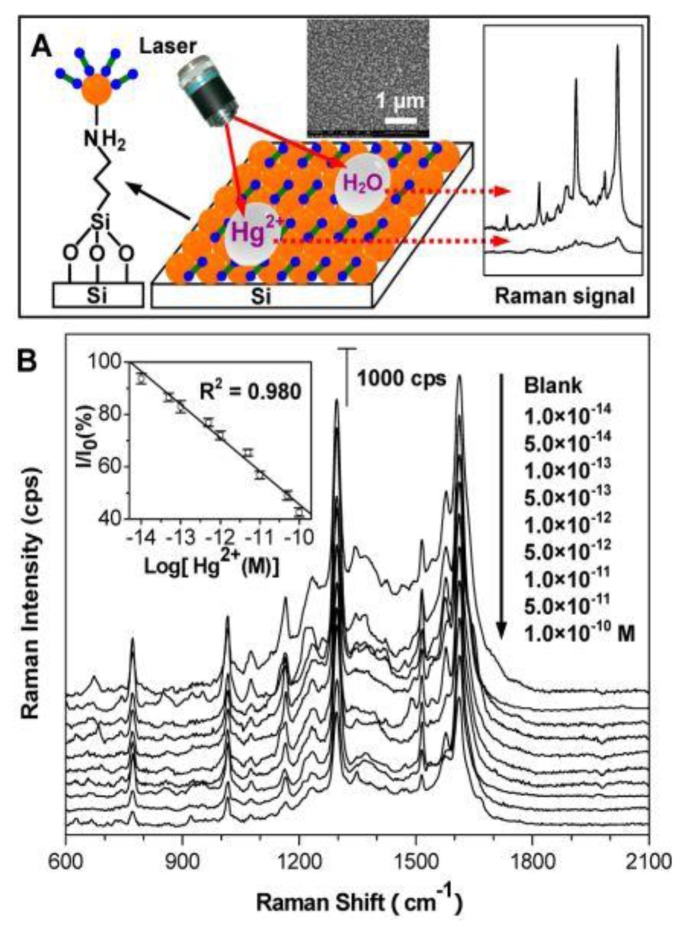
(**A**) Schematic drawing for direct detection of Hg^2+^ with the SERS chip fabricated by the assembly of Au@Ag NPs on a silicon wafer; (**B**) Evolution of SERS spectra of Dpy with the addition of anaqueous Hg^2+^ droplet. The inset is the linear correlation of Raman intensity (at 1614 cm^−1^) with the logarithm of Hg^2+^ concentrations from 1.0 × 10^−14^ to 1.0 × 10^−10^ M (reprinted with permission from [121]. Copyright (2013) American Chemical Society).

**Figure 16.**
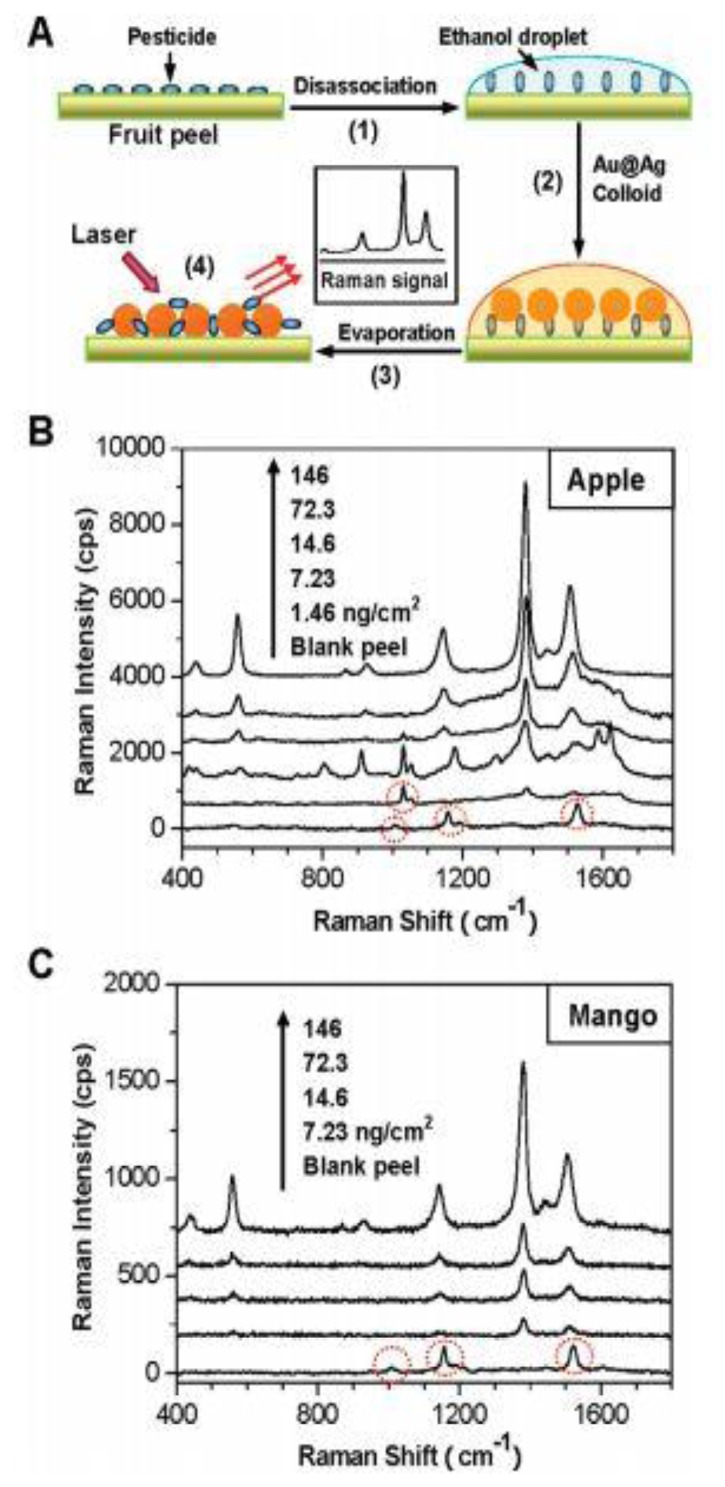
(**A**) Schematic drawing for the direct detection of pesticide residues at fruit peels by the SERS spectroscopy using Au@Ag NPs as Raman amplifier, and the SERS spectra of (**B**) apple and (**C**) mango peels spiked with thiram by the enhancement of Au@Ag NPs colloid that was cast onto the surfaces of the fruit peels. All Raman spectra were recorded with a 532 nm laser with 5 mW power and 10× objectives (reprinted with permission from [124]. Copyright (2012) American Chemical Society).

Metal ions do not have Raman signals, which have to be detected by indirect methods leading to inefficiency. To solve the problem, many efforts have been devoted to find ways to detect metal ions (such as Hg^2+^, Cu^2+^) in a simple, rapid and ultrasensitive way [118–120]. However, we found that the detection limitation of Hg^2+^ was 10^−12^ M by using a single-metallic nanostructures as sensor. To further decrease the detection limitation, Du *et al.* [121] applied a SERS chip constituted of Au@Ag core-shell nanoparticles with an organic ligand (Dpy) in the detection of Hg^2+^ as shown in Figure 15a. When the water droplet containing Hg^2+^ was added onto the chip, the Raman signals of Dpy obviously quenched due to the formation of Hg^2+^-Dpy complex. The results in Figure 15b show that even 10^−14^ M Hg^2+^ can been detected due to the more excellent SERS properties of Au@Ag core-shell nanostructures. In addition, this approach just needs 4 min and 20 μL of sample. Therefore, Au@Ag core-shell nanostructures can be used for the ultrasensitive detection of trace Hg^2+^. Similar to Hg^2+^, Cu^2+^ has paramagnetic properties which can transfer electrons or energy resulting in photoluminescence (PL) quenching of Au@Ag core-shell nanoparticles. Gui *et al.* [122] first employed Au@Ag core-shell nanoparticles in the development of a PL sensor for the detection of Cu^2+^. As the concentration of Cu^2+^ increased, the PL spectra of Au@Ag core-shell nanoparticles dropped dramatically, so we can analyze Cu^2+^ according to the intensity of PL spectra. In addition to metallic ions analyses, biomolecules and organic molecules have attracted much attention. Panfilova *et al.* [123] first studied the application of multicolor Au@Ag core-shell nanoparticles in a multiplexed solid-phase dot immunoassay. During the transformation process from silver nanocubes to Au@Ag core-shell nanoparticles, the color varied from yellow to blue with the shift of the SPR peak from 450 to 700 nm. Then different color nanoparticles were conjugated with different immuno gamma globulin molecular probes. As a result, when these colorless anti-rat rabbit antibodies were stained by functional molecular probes, they showed the corresponding colors, which can be regarded as detection basis of immunoassay. Liu *et al.* [124] reported the shell thickness-dependent Raman enhancement of Au@Ag core-shell nanoparticles for the identification and detection of pesticide residues at various fruit peels, where it was first proposed that the huge Raman enhancement was contributed by individual Au@Ag NPs rather than aggregated Au@Ag core-shell nanoparticles with hotspots among the neighboring NPs. Figure 16 illustrates a schematic drawing (A) for the direct detection of pesticide residues at fruit peels by SERS spectroscopy using Au@Ag NPs as Raman amplifier, and the SERS spectra of (B) apple and (C) mango peels spiked with thiram by the enhancement of Au@Ag NPs colloid that was cast onto the surfaces of the fruit peels.

In the research of Zheng *et al.* [125], Au@Ag core-shell bipyramids was used for a fast and reproducible determination of thiram, which provided satisfactory results for the dectection of thiram in environmental water samples. Furthermore, bifunctional core-shell particles have multiple functions. For example, FePt@Ag core-shell nanostructures not only have excellent SERS properties, but also high saturation magnetization [126]. Although Fe and Pt belong to the transition-metals which have rather weak SERS signals [127], they can achieve high SERS activity by borrowing SERS activity from silver or gold nanostructures which can create long-range effect of the very strong electromagnetic field to enhance the Raman scattering of adsorbents. The magnetic–plasmonic FePt@Ag NPs have potential applications in biomedicine.

## 6. Conclusions

In this review, we present double reductant methods, etching techniques and construction of core-shell nanostructures routes which can synthesize novel shape-controlled silver nanostructures. Various silver nanostructures have been prepared by using the three methods. The double reductant method takes full advantage of different favorable facets of nanostructures produced in different reductants. In this case, complex nanostructures which are not easily prepared by one-step methods can be prepared in high yield with mono-dispersed sizes. The etching technique can synthesize hollow nanostructures with tunable silver shell thickness which can be used for controlling their LSPR. Moreover, etching technique can also increase the roughness of the surface of silver nanostructures, significantly increasing SERS enhancement and LSPR effect. The construction of core-shell nanostructures can not only control their shapes and sizes well, but also exhibit more excellent optical properties than that of individual metal nanostructures. Finally, we introduce applications of silver nanostructures produced by the three methods. Because of their excellent optical properties, such as LSPR properties and SERS effect, these silver nanostructures have potential applications for the development of plasmonic nanodevices and detection.

## Figures and Tables

**Table 1.** Comparison of physical constants between the core metal and the shell metal in various combinations of binary metal nanocrystals (reprinted with permission from [107]. Copyright (2008) American Chemical Society).

**Core-Shell**	**Atomic Radius r_core_** **vs r_shell_**	**Bond Dissociation Energies**	**Electronegativiy (Paulings)**	**Experimental Observation of Epitaxial Growth**
Pt @ Au	small	high	low	no
Pt @ Ag	small	high	high	no report
Pt @ Pd	large	high	high	yes
Au @ Pd	large	high	high	yes
Au @ Ag	equal	high	high	yes
Au @ Pt	large	low	high	no
Ag @ Pd	large	high	low	no
Ag @ Au	equal	low	low	no
Ag @ Pt	large	low	low	no
Pd @ Au	small	low	low	no
Pd @ Ag	small	low	high	no report
Pd @ Pt	small	low	low	no report
